# Lectures on Scarlet Fever

**Published:** 1851-06

**Authors:** Caspar Morris

**Affiliations:** Late Lecturer on Practical Medicine in the Philadelphia Medical Institute


### MEDICAL EXAMINER,

AND

RECORD OF MEDICAL SCIENCE.

NEW SERIES.—NO. LXXVIII.—JUNE, 1851.

### ORIGINAL COMMUNICATIONS.

*Lectures on Scarlet Fever.* By CASPAR MORRIS, M. D., late Lecturer on Practical Medicine in the Philadelphia Medical Institute.

### LECTURE V.

Such are the phenomena which usher in, and mark the progress of, scarlet fever; and such the primary results of its impression on the human system. We are now prepared to arrive at some conclusion as to its character, which may be thus expressed.

Scarlet fever is a febrile disease, dependent on a specific miasm, resulting from unknown external influences; yet capable of reproduction, and diffusing itself, both by direct contagion and by the establishment of a peculiar condition of the atmosphere within limited districts: the susceptibility to the influence of this cause varies in different individuals and at different periods of life, and is generally exhausted by one attack: the morbid impression is made on the nervous system, and transmitted to the circulating fluids through which every part of the organization becomes subject to the influence, while the principal localization of diseased action is in the capillaries of the skin and mucous membrane, especially that which covers the pharynx: it has a fixed duration of from 120 to 160 hours, at the close of which period the disease has a natural solution, unless the disturbance of the functions has been so great as to destroy life, or local lesions have been produced which maintain the irritative action.

We now proceed to the consideration of certain results of this impression, which are more durable than the disease itself; quite distinct in their character, equally, perhaps more, to be deprecated than the primary disease.

These are generally known as the *sequelæ*, and as such, have been occasionally referred to in our previous lectures. They may be divided into two classes. 1st, Those which appear to be a mere continuation of the local lesions developed during the course of the disease, which are confined to the same vicinity; sloughing and ulceration of the throat, diseases of the ear, often extending to the bony structure, and abscesses about the neck.

It can scarcely be necessary to add any thing to the remarks already made on the subject of the affection of the throat, when treating of that symptom in the description of the anginose and malignant forms of the disease. It occasionally happens, however, that the ulcerative process set up from the very commencement of the case, continues long after the scarlet fever has run through its regular stages; and extends itself upwards into the posterior nares, or downward into the pharynx, or even invades the larynx.

It may, however, be well to inform you, that a question has been raised, whether there is ever an actual solution of continuity of the parts about the throat. Of this there can be no doubt on the part of those who have seen the anginose and malignant forms in their greatest intensity. I have known the entire destruction of the soft palate, and extensive sloughing of the adjacent parts even where recovery has followed.

Disease of the ear may either occur during the course of the primary disease and be prolonged after that has reached its termination, or may be first manifested even some weeks after the scarlet fever has seemingly disappeared. It is sometimes ushered in by a rigor and deep seated pain, followed by purulent discharge, and at others, is first manifested by discharge from the external meatus of a thin apparently serous fluid, which soon takes on the appearance of unhealthy pus.

The cases which thus manifestly originate in the membrane lining the external meatus generally yield speedily to appropriate treatment; while those which are dependant either on the transmission of the inflammation along the Eustachian tube, or in which that process has its commencement in the internal ear, are of a much more serious character. In either case permanent deafness may result; if both ears are affected, reducing the child to the condition of a mute. I had a case some years since in the person of a fine healthy boy of about eight years old, in which the primary symptoms were all of the most aggravated character. The swelling of the throat and ulceration of the mucous membrane of the pharynx and nares were very extensive. As the primary disease subsided the irritative action of these local lesions took its place, prolonging the duration of the case for many weeks. Finally, a chill, followed by intense febrile reaction and extreme pain in the ear, announced the extension of the disease to the internal ear. Stupor, interrupted with violent shrieks, like those which occur in acute meningitis, supervened: suppuration was established, and the matter found its way to the surface by the external meatus, and by an abscess over the mastoid process, evidently connected with the cells of that portion of the temporal bone. Caries of the bone followed, which continued for several years, and though the general health of the child was in great measure re-established, nothing had any influence in arresting the disease of the bone, which continued to extend itself until ultimately it reached the membranes of the brain, and he died of acute meningitis.

Croup may also occur as a sequela of scarlet fever, as well as during the primary stages of the disease; in either case a most formidable complication. Some years since one of my patients survived an attack of scarlet fever in which every complication and sequela of the disease appeared to conspire for her destruction. The disease was ushered in by convulsions. There was extensive sloughing of the soft palate, and then as late as the tenth day the hard croupal cough gave evidence of the invasion of the larynx. I attempted the application of the solid nitrate of silver to the part, when to my infinite dismay a large portion was broken off by the child in its struggles and swallowed. I said nothing of the accident to the parents at the moment, but calling for the salt cellar, promptly administered a strong solution of the muriate of soda. This not only decomposed the nitrate of silver but acted as a speedy and efficient emetic, and very probably saved the child's life, by the vomiting induced, as to my great surprise it recovered.

Still more frequently, the lymphatic glands of the neck become implicated, and serous effusion into the cellular tissue beneath the jaw impedes the deglutition and respiration, and either accelerates the fatal catastrophe, or produces the formation of large collections of pus, which may give rise to hectic irritation, and result in death after prolonged suffering.

The 2d class embraces lesions, which are more general in their character; among which the first place in importance as well as in order of time, must be assigned to that destruction of the epidermis by the cutaneous inflammation which gives rise to the process of *desquamation*, to which reference has been already made. It may be indeed a question well worthy investigation, how far other sequelæ, inflammation of the kidneys, and dropsical effusions, and inflammation of the membranes of the heart, may be dependant on this interruption of the functions of the skin. Certain it is, that all these sequelæ are observed to follow more frequently those cases in which the affection of the skin has been most decided. Some authors make a contrary assertion; but my own observation is very positive on this point. Thus in the malignant and irregular forms of the disease, the mortality is greatest in the first week, or results from the injury done to the brain or nervous system, by the direct onset of the disease. While in those mild cases of the simple form in which there is abundant efflorescence, with high febrile reaction, death, if it occur at all, is produced by the dropsical effusion, or some of the other secondary affections, as those of the heart or brain. When the rash has been very vivid, and the heat and dryness of the skin corresponding, the destruction of the epidermis is most decided; and most writers agree in testimony, that in such cases, there is more reason to apprehend the occurrence of serious sequelæ, while in those cases in which the rash does not appear, it is but slight, or recedes soon after its appearance; the functions of the skin being less disturbed, the convalescence is less frequently interrupted by the occurrence of dropsical effusions, or of inflammation of the membranes of the heart, which prove the most fertile sources of danger after the first stage of the disease has been safely passed through. You must also bear in mind the fact that the disease of the skin is more strongly marked in the simple and anginose than in the malignant or irregular forms, and all writers coincide in the declaration that the sequelæ are more usual in the mild and regular, than in the severe and irregular cases.

The importance of the skin, and the influence upon the whole economy of the mode in which its functions are performed, cannot be too strongly impressed on your minds. We all are conscions of the effect on our own sensations arising from the arrest of those functions, by whatever cause it may be produced; and you know every feeling of discomfort is only an indication of a deviation from health. This, however, does not make so strong an impression on your minds as the facts which are proven by observation on the results of the healthy action; when for example, you estimate the influence on the circulating fluids which must be produced by the twenty-eight miles of "perspiratory tubing," with its seven millions of pores opening through the epidermis, discharging daily two ounces of excrementitious matter, and not less than thirty ounces of watery vapor, you can better appreciate the effects which must result from the entire interruption of this excretion, produced by the drying of the cuticle through which those pores are transmitted: nor must you forget that this excretory process is not the only one which is going on through cutaneous agency. From the teacher of Physiology you will learn how large is the amount of influence on the blood, subsidiary to that of the lungs, exerted by the direct imbibition of oxygen through the skin. Now if you bear in mind the activity of the vital processes, and that no one of them is needless, but each absolutely essential to the other, and to the integrity of the whole organization; and then remember that by the destruction of the cuticle not only the depurative action is arrested, but an obstruction placed on one channel, by which the vitalizing influence of oxygen is introduced, you will be better prepared to appreciate the condition of the blood, and the consequent danger to the patient, in scarlet fever, from this cause. It is, moreover, a fact well established by the observation of surgeons, that in burns and scalds, the danger is proportioned less to the depth of the injury and the intensity and duration of the heat applied, than to the extent of surface involved, thus affording additional evidence of the importance to the system of the cutaneous function. Nor must you overlook the fatal results which have been produced, on animals of a lower grade, by coating the skin with some impermeable material. Now in this disease the whole surface is affected, and more decidedly than in any other of the exanthemata, except confluent small pox. In rubeola there are patches of healthy skin; in erysipelas the extent of surface involved at one time is much smaller, and neither of these diseases leaves the dead cuticle to impede the normal action of the new. You will not, therefore, be surprised that when treating of the inferences, as to the final result, to be drawn from the symptoms of the disease, I cautioned you against the common error, of supposing that the freeness and extent of the cutaneous affection was a favorable sign; and will understand why I thus draw your attention to the desquamatory process and place it first among the sequelæ of the disease; and will also be the better able to comprehend why the mortality is often greater in the secondary than the primary stages. So decidedly is this the case, that common observation has taught it. When on one occasion I assured an intelligent mother of a large family, several of the children having been attacked simultaneously, that the crisis of the disease was passed, she replied: "well, then this is the period of danger; Mr. ———, who lost several children with it, told me that they all died just as the doctor told him they were getting well."

This, then, is the period in which your anxieties should be especially active and your care most vigilant; and the danger to the patient is connected with, if not wholly dependent upon, the interruption to the healthy action of the cutaneous emunctories. The process of casting off the cuticle, which we observe, and to which this term *desquamation* is applied, is not itself a diseased process, but the effort to get rid of an impediment to the normal action of the system. Commencing from the time at which the cuticle is destroyed in favorable cases, in others it is postponed, sometimes even for a fortnight. You will always find the peril proportioned to the tardiness with which it is accomplished; and until it is completed, you will find your patients depressed by the poisoned condition of the blood, and liable to the sudden development of cerebral disease or inflammation in some organ essential to life. But the external cuticle is not the only organ for exhalation, the action of which is in a similar manner suspended or destroyed by the disease. You are of course aware from observation on your own person, and have been taught to apply this observation to its relation to disease, that wherever any influence from *without* arrests the cutaneous transpiration, a corresponding increase is found in the watery constituent of the secretion from the kidneys. This is not the case, however, in diseases where the same cause certainly operates equally on both kidneys and skin, and produces a similar result in each. Hence you will find, that though the amount of urine has been but little diminished during the four or five first days of scarlet fever, it is either wholly arrested or becomes very scant about the same time that the functions of the skin are most impeded. The same is true in other febrile diseases, in most of which, you will find the excretions through the skin and kidneys are not antagonistic as in health, but are either suppressed or increased in freeness under the influence of some common cause. The occurrence simultaneously of increased discharges from both organs is noted as marking the crisis of febrile diseases. Now it is not at all a forced idea that the same diseased action may be established in the Malpighian bodies and epithelium of the kidneys, as is observed on the external tegument; and it is well known that the flow of urine is always much diminished about the time at which the process of desquamation is at its height, and I believe epithelial scales are found at this juncture abundantly in the urine. The facts observed therefore, confirm the suspicion which might be entertained from reasoning only, that there is such a connexion beween the diseased condition of the skin and kidneys, in their influence upon the other sequelæ of scarlet fever, as justifies the relation which I have ventured to suggest before; and indicate the latter organs as equally interested with the skin in the production of some of the results which will claim your attention immediately. Whether the diseased condition of the kidneys be dependant on the interruption of the functions of the skin, or both organs are simultaneously affected by one common agency, I am not yet prepared to decide positively. Certain it is, that dropsical effusions occur in a very large proportion of cases of regular scarlet fever, frequently, in despite of all precautions, always if care be not taken to prevent exposure to atmospheric vicissitudes or errors of diet. The more regular the course of the case, the more vivid and abundant the eruption, the more certainly may this desquamation be expected, and its consequences, dropsical effusion, be looked for.

The external cellular tissue affords the most common seat for the serous effusion. It may take place, however, into any or all the cavities of the body from the ventricles of the brain or pericardial sac, or cellular structure of the lung, to the tunica vaginalis testis.

The actual effusion of fluid is generally preceded, a longer or shorter time, sometimes only four or five hours, in other cases, for a day or two, by feelings of languor and drowsiness, loss of appetite, nausea and vomiting, coated tongue, and great heat and dryness of the skin: the pulse is tense and corded, with a return of the frequency if that had subsided. From the end of the first week to the beginning of the third, is the period at which these symptoms are most frequently developed. Though no amount of precaution can wholly remove the liability to the result, it is by no means uncommon to find it brought about by improper exposure to atmospheric vicissitudes, or some error in diet; and it is to the neglect of care in these points, resulting from the idea that the disease has been too mild to require it, we may ascribe the greater proportionate frequency of dropsy after the mildest cases of simple scarlet fever.

The present will probably be as appropriate a place as any that may offer to impress on you the importance of enforcing the greatest care with regard to this exposure, and indulgence of appetite, since it is sometimes very difficult to make those who have not purchased their own experience by a severe lesson fully aware of its necessity.

In the year 1830, I was sent late at night, by the late

Dr. Dewees, to see the family of an officer of the Navy, in which five of the children had been under the care of another physician, with mild scarlet fever. He had seen them in the morning, pronounced them well, and had given his consent to their being allowed the free range of the house and the usual diet of the family. When I reached the house, two of the children were already dead from convulsions, and the others were saved with difficulty, having fever followed by anasarca, depending, in all probability, on inflammation of the kidneys, the result of the exposure of the day. No examination of the bodies was permitted, but the deaths were undoubtedly owing to effusion of fluid within the cranium. Dr. Chapman describes similar results as having occurred under his own observation.

In another instance I was attending a child from South Carolina at lodgings here, which was carried happily through a mild attack, with but little treatment except of a preventive character. An older daughter of about 18 was taken sick after I had ceased my attendance in the first case. The symptoms were so benign, and the treatment I had adopted had been apparently so nugatory, that the family were induced to believe the disease was but little to be dreaded, and required no care whatever. They, therefore, did not seek medical advice in the second case, and permitted her to pursue her usual habits, as regards diet and exposure; at the end of a fortnight I was sent for to prescribe for general anasarca for which they were at a loss to account. Warned by these and similar instances, some of which I shall refer to when treating of other sequelæ, I am always earnest in my endeavors to impress on the parents of scarlet fever patients, the great importance of care on these points, until the entire process of desquamation has been completed; and this care is more especially important, at the very juncture when it is least likely to be exercised, just at the close of the process, when the condition of the blood caused by the arrest of the secretion from the skin and kidneys is at its worst point, and the new cuticle most tender and liable to the impression of cold air. The effusion is generally first observed about the face and hands. The complexion suddenly assumes a less healthy hue, resembling that of anemia, the eye lids are observed to be somewhat puffy, and upon attempting to flex the fingers they are found to be stiffened. The trunk and upper parts of the lower limbs next assume the appearance of disease, and on feeling these parts they are sensibly harder to the touch than natural, and there is often some complaint of tenderness on pressure, which, moreover, does not leave the indentation found commonly in anasarca. All these circumstances agree in the indication that the skin itself is in fault, and that the disease is not one of mere infiltration, caused by some affection of the viscera, or destruction of the crasis of the blood. The suddenness with which this swelling follows exposure, also confirms the same view, as I have known it to be manifested almost immediately on the return from a drive, or exposure at the door or window, or other similar imprudence. The bowels are generally costive at this time, and even though the secretion of urine may not before have been so much diminished as to arrest attention you will now find, if you make enquiry, that it is very scanty, high colored, and turbid. Those who have made critical examination of this fluid under these circumstances report it to contain blood, and albumen, and to be very acid. At one time this was thought to indicate the existence of the incipient stage, that peculiar condition of the kidney, found in cases of what is known as Bright's disease. This is, however, an error. The presence of albumen in the urine as indicated by the deposit of that substance coagulated by heat or nitric acid, is often dependant on simple inflammation or perhaps congestion only of those organs, and this is undoubtedly the case in the instances under consideration. In Bright's disease the quantity of the urine is increased, its specific gravity diminished, its color light: in all these respects presenting a marked contrast with the condition of the secretion in the dropsy following scarlet fever. The examination of the bodies of those who have died of scarlet fever, as reported by MM. Rilliet and Barthez, proves that no such disorganization as that known to produce Bright's disease exists. It is true these authors do not indicate how many of their fatal cases had progressed to the period in which dropsy is developed, but they say that "in no case were the kidneys enlarged or pallid, though sometimes red or congested." There is no disease in which we stand more in need of accurate, well digested observations of post-mortem examinations. This results from the fact that the mortality generally occurs in private practice, and the distress of parents and friends prohibits the physician from proposing anything which would aggravate their sorrow.

Anasarca is the form in which the dropsical effusion most generally presents itself. If it were the only one, the previous diseased condition of the cutaneous capillaries, and the generally acknowledged dependance of the disease on the impression of cold on the surface, would justify the belief which has been expressed by some authors that "it arises from increased action in the sanguiferous system."* Next to the cellular tissue beneath the skin in liability to this effusion, I should place the pleural sac or the pulmonary tissue; and after this in order of frequency, the ventricles of the brain and the pericardium, and I have no doubt that sudden death is caused in many cases by these latter lesions. An instance of this occurred in my own practice. An infant had been carried out by its nurse, apparently in perfect health. It was brought home in a state of collapse, and died in a few hours without reaction. Within a few days another child in the same family sickened with scarlet fever, and had a mild anginose attack with an abundant eruption. It passed through the first week safely, and was apparently convalescent, when an anxious grandmother, confident that it stood in need of more nourishment than I had allowed to be given, fed it largely with cakes, ice cream, and animal food. At the end of 48 hours of this injudicious treatment, the child was seized with general anasarca, with dry cough, dyspnœa to such a degree as prevented the child from lying down, and death speedily ensued. In this case there was pulmonary œdema and effusion into the pleura and pericardium. Dr. Hamilton reports three similar cases among the boys in a school in England, in which death took place within thirty-six hours, the symptoms indicating effusion in the ventricles of the brain, and the cavities of the thorax and abdomen, as well as in the cellular tissue generally. Cerebral effusion is certainly the most formidable of the sequelæ of scarlet fever, though happily less frequent than the anasarca. It is indicated by the occurrence of stupor, or violent headache and vomiting. The pulse is slow, one or both pupils dilated. Vision

*Tweedie, Cyclop. Pract. Med.

is impaired or even lost entirely, and there may be strabismus, or even general convulsions, or paralysis. The countenance is at the same time bloated, often excessively so.

Endo-cardial inflammation is another of the sequelæ of scarlet fever of a most formidable character, which you will find mentioned by most authors. It has only once met my notice. This was in the case of a lad of 12 years old. Scarlet fever in its mildest form had passed through a family of six children, of which he was the oldest and last taken. The most rigid attention to diet, and the greatest care to avoid exposure, had been practised; my attendance had been prolonged to six weeks, and as convalescence was fairly established, I had called in the evening to take my leave, and sat till a late hour. In parting I expressed my gratification at the result, for I can assure you, you have great cause for thankfulness when this disease assumes a form so benign as to pass through a family of that size, and leave the circle unbroken. The reply of the mother, was the expression of her fear that the peril was not yet entirely passed. The hearty laugh of the one that had last been sick, which was just then heard from the nursery, might have induced me to ridicule such apprehensions had I not known the treacherous character of the disease. Scarcely three hours had elapsed when I was summoned to see him with great dyspnœa, tossing about the bed unable to lie down, with a small tense pulse and hot skin. Bleeding, leeching, blistering, calomel, digitalis and colchicum, aided by the most anxious and affectionate nursing and rigid abstinence, carried him through this attack, but left him with an hypertrophied condition of the left ventricle, and disease of the valves, from which he has, however, since recovered.

*Rheumatic* pains, with fever, are of very frequent occurrence during the convalescence from this disease. It is often, though not always the result of premature exposure, and like the dropsical effusion, is more likely to occur in mild than grave cases. A fine healthy child was spending the summer months in the country where scarlet fever had not before prevailed. He had an eruption with febrile symptoms, but of so mild a character that the physician in attendance, remarked, he might have had scarlet fever, but that there was not sufficient disturbance of the child's health to correspond with the amount of eruption, and his ideas of the disease. The little fellow was allowed to pursue his usual amusements without interruption. At the end of a week he complained that his "legs were too long for him," that he was "tired," became feverish and languid, refused food, lost the power of using his lower extremities, and died. This was an extreme case; more generally some time during the second week, there is a return of the fever, the pulse quick and corded, and the skin hot and dry; the child complains of pain in the legs, and cries when any attempt is made to move it. This state continues two or three days, and then yields to proper treatment.

Some authors speak of purulent deposits in the joints. This is a result which has never fallen under my notice.

Diarrhœa, when it occurs, is to be ascribed to the irritation of the putrid colluvies swallowed from the ulcers in the throat, rather than to any direct influence of the cause of the disease on the mucous membrane of the alimentary canal.

(To be continued.)

